# Survival benefit of radiotherapy after surgery in de novo stage IV breast cancer: a population-based propensity-score matched analysis

**DOI:** 10.1038/s41598-019-45016-2

**Published:** 2019-06-12

**Authors:** Yi-Jun Kim, So-Youn Jung, Kyubo Kim

**Affiliations:** 1 Center for Precision Medicine, Seoul National University Hospital, Seoul, Republic of Korea; 2 Department of Radiation Oncology, Ewha Womans University College of Medicine, Seoul, Republic of Korea; 3 Department of Radiation Oncology, Graduate School of Medicine, Ewha Womans University, Seoul, Republic of Korea; 4 Center for Breast Cancer, National Cancer Center, Goyang, Gyeonggi Republic of Korea

**Keywords:** Breast cancer, Outcomes research

## Abstract

The survival benefit from radiotherapy in stage IV breast cancer has not been fully evaluated. We investigated the survival benefit of radiotherapy after surgery in *de novo* stage IV breast cancer. Using a population-based database (the Surveillance, Epidemiology, and End Results database 18, 2010–2013), patients diagnosed with *de novo* stage IV breast cancer were divided into those undergoing surgery alone (no-radiotherapy group) and those undergoing surgery followed by radiotherapy (radiotherapy group). After propensity-score matching (PSM), the cancer-specific survival (CSS) rates were estimated. Multivariate analysis was performed to evaluate the prognostic value of radiotherapy on survival. After PSM, the 3-year CSS rates in the no-radiotherapy (n = 882) and radiotherapy (n = 882) groups were 57.1% and 70.9% (P < 0.001), respectively. On multivariate analysis, radiotherapy after surgery was a significant prognosticator (hazard ratio [HR] 0.572; 95% confidence interval [CI] 0.472–0.693, P < 0.001). Regardless of surgery type and lymph node involvement, the radiotherapy group showed significantly higher CSS rates. For patients who survived six months or more, radiotherapy after surgery demonstrated favorable prognosis compared to surgery alone (HR 0.593; 95% CI 0.479–0.733, P < 0.001). In conclusion, radiotherapy after surgery increased CSS rates in *de novo* stage IV breast cancer compared to surgery alone.

## Introduction

Metastatic breast cancer is a systemic disease^1^ and local treatments such as surgery or radiotherapy that are effective for early breast cancer are not sufficient for locally advanced or metastatic disease. This realization derived the development of systemic management in breast cancer. Chemo-, hormone, and targeted therapies have shown remarkable survival benefits in locally advanced and even metastatic breast cancers, and survival has been extended^2–4^. However, as advances in systemic therapy have extended the life expectancy of metastatic breast cancer patients, the utility of local treatments has become a new question in *de novo* stage IV breast cancer patients^5^.

There is no generalized category I treatment for management of *de novo* stage IV breast cancer. The National Comprehensive Cancer Network (NCCN) guidelines recommend both local treatment and systemic therapy for patients with stage IV breast cancer, without prioritization^6^.

Local treatment in stage IV breast cancer has several mechanisms of action. Local treatments reduce tumor burden^7^, eliminate cancer stem cells^8^, reverse tumor-induced immunosuppression^9^, decrease clonal heterogeneity^10^, and avoid self-seeding of the primary tumor, which is correlated with distant metastasis^11^. In clinical practice, however, the survival benefit of local treatments in *de novo* stage IV breast cancer is controversial. Although retrospective studies have shown that local treatments increase survival^12–16^, recent randomized controlled trials that investigated the survival benefit of surgery of the primary site revealed mixed conclusions^17–20^.

Local treatment in *de novo* stage IV breast cancer usually refers to the surgery of primary sites^18^. Unlike surgical treatment in *de novo* stage IV breast cancer, the survival benefit of radiotherapy has rarely been investigated^21,22^. One study showed that exclusive loco-regional radiotherapy can improve survival compared with no local treatment^23^. However, this study was not a comparison between only surgery and combination of surgery and radiotherapy.

To date, the impact of radiotherapy after surgery on the survival for *de novo* stage IV breast cancer patients in the literature is ambiguous, and no consensus exists^23–26^. In this study, we investigated the survival benefit of radiotherapy after surgery in *de novo* stage IV breast cancer using a population-based database including information on metastatic sites (the Surveillance, Epidemiology, and End Results [SEER] database 18, 2010–2013).

## Results

### Patient characteristics before and after propensity-score matching

There were 1325 patients in the no-radiotherapy group (only surgery of the primary site) and 882 in the radiotherapy group (surgery of the primary site followed by radiotherapy of the primary site and/or metastatic sites). The patient characteristics are summarized in Table 1. Although the exact site of radiotherapy was unavailable from the SEER database, there were significant differences between the two groups. Patients treated with radiotherapy were more likely to be young (under 60, 47.1% in the no-radiotherapy group vs. 57.0% in the radiotherapy group, P < 0.001), be hormone receptor-positive (64.6% vs. 73.5%, P < 0.001), have bone metastasis (52.5% vs. 62.8%, P < 0.001), be treated with breast-conserving surgery (31.5% vs. 37.2%, P = 0.005), and be married (44.8% vs. 49.5%, P = 0.025). Patients who did not receive radiotherapy had more lung (30.3% vs. 17.2%, P < 0.001) and liver metastases (23.5% vs. 15.1%, P < 0.001) and multiple metastases (19.3% vs. 14.4%, P = 0.003).

**Table 1:** Characteristics of stage IV breast cancer patients before and after PSM.

Characteristics	Before PSM					After PSM				
	Surgery only		Surgery and radiotherapy		p*	Surgery only		Surgery and radiotherapy		p*
	n = 1325		n = 882			n = 882		n = 882		
	no.	(%)	no.	(%)		no.	(%)	no.	(%)	
**Age**										
<50	294	(22.2)	244	(27.7)	<0.001	238	(27.0)	244	(27.7)	0.247
50–59	330	(24.9)	258	(29.3)		246	(27.9)	258	(29.3)	
60–69	326	(24.6)	230	(26.1)		215	(24.4)	230	(26.1)	
≥70	375	(28.3)	150	(17.0)		183	(20.7)	150	(17.0)	
**Race**										
White	1019	(76.9)	668	(75.7)	0.481	665	(75.4)	668	(75.7)	0.361
Black	203	(15.3)	137	(15.5)		152	(17.2)	137	(15.5)	
Others	98	(7.4)	76	(8.6)		62	(7.0)	76	(8.6)	
Unknown	5	(0.4)	1	(0.1)		3	(0.3)	1	(0.1)	
**T stage**										
≤T1	168	(12.7)	110	(12.5)	0.020	126	(14.3)	110	(12.5)	0.428
T2	472	(35.6)	338	(38.3)		334	(37.9)	338	(38.3)	
T3	256	(19.3)	151	(17.1)		168	(19.0)	151	(17.1)	
T4	371	(28.0)	265	(30.0)		235	(26.6)	265	(30.0)	
Unknown	58	(4.4)	18	(2.0)		19	(2.2)	18	(2.0)	
**N stage**										
N0	251	(18.9)	131	(14.9)	0.011	165	(18.7)	131	(14.9)	0.232
N1	491	(37.1)	316	(35.8)		319	(36.2)	316	(35.8)	
N2	251	(18.9)	183	(20.7)		169	(19.2)	183	(20.7)	
N3	291	(22.0)	234	(26.5)		212	(24.0)	234	(26.5)	
Unknown	41	(3.1)	18	(2.0)		17	(1.9)	18	(2.0)	
**Histology**										
Invasive ductal carcinoma	975	(73.6)	680	(77.1)	0.306	649	(73.6)	680	(77.1)	0.224
Invasive lobular carcinoma	121	(9.1)	73	(8.3)		97	(11.0)	73	(8.3)	
Others	208	(15.7)	117	(13.3)		122	(13.8)	117	(13.3)	
Unknown	21	(1.6)	12	(1.4)		14	(1.6)	12	(1.4)	
**Grade**										
Well differentiated	74	(5.6)	56	(6.3)	0.024	57	(6.5)	56	(6.3)	0.930
Moderately differentiated	415	(31.3)	303	(34.4)		293	(33.2)	303	(34.4)	
Poorly differentiated	722	(54.5)	472	(53.5)		482	(54.6)	472	(53.5)	
Undifferentiated	10	(0.8)	10	(1.1)		7	(0.8)	10	(1.1)	
Unknown	104	(7.8)	41	(4.6)		43	(4.9)	41	(4.6)	
**IHC subtype**										
HRc+/HER2−	653	(49.3)	489	(55.4)	<0.001	476	(54.0)	489	(55.4)	0.138
HRc+/HER2+	203	(15.3)	160	(18.1)		130	(14.7)	160	(18.1)	
HRc−/HER2+	132	(10.0)	64	(7.3)		76	(8.6)	64	(7.3)	
HRc−/HER2−	236	(17.8)	126	(14.3)		151	(17.1)	126	(14.3)	
Unknown	101	(7.6)	43	(4.9)		49	(5.6)	43	(4.9)	
**Bone metastasis**										
No	630	(47.5)	328	(37.2)	<0.001	324	(36.7)	328	(37.2)	0.844
Yes	695	(52.5)	554	(62.8)		558	(63.3)	554	(62.8)	
**Lung metastasis**										
No	923	(69.7)	730	(82.8)	<0.001	735	(83.3)	730	(82.8)	0.751
Yes	402	(30.3)	152	(17.2)		147	(16.7)	152	(17.2)	
**Liver metastasis**										
No	1014	(76.5)	749	(84.9)	<0.001	731	(82.9)	749	(84.9)	0.244
Yes	311	(23.5)	133	(15.1)		151	(17.1)	133	(15.1)	
**Combination of metastatic sites**										
Bone metastasis only	477	(36.0)	433	(49.1)	<0.001	428	(48.5)	433	(49.1)	0.812
Lung metastasis only	228	(17.2)	73	(8.3)	<0.001	71	(8.0)	73	(8.3)	0.862
Liver metastasis only	149	(11.2)	63	(7.1)	0.001	68	(7.7)	63	(7.1)	0.650
Other metastasis only	215	(16.2)	186	(21.1)	0.004	176	(20.0)	186	(21.1)	0.555
Bone and lung metastases	136	(10.3)	73	(8.3)	0.118	67	(7.6)	73	(8.3)	0.597
Bone and liver metastases	124	(9.4)	64	(7.3)	0.083	74	(8.4)	64	(7.3)	0.375
Lung and liver metastases	80	(6.0)	22	(2.5)	<0.001	20	(2.3)	22	(2.5)	0.755
Lung and/or liver metastases	633	(47.8)	263	(29.8)	<0.001	278	(31.5)	263	(29.8)	0.439
**Multiple sites of metastases**										
No	1069	(80.7)	755	(85.6)	0.003	743	(84.2)	755	(85.6)	0.425
Yes	256	(19.3)	127	(14.4)		139	(15.8)	127	(14.4)	
**Surgery**										
Breast-conserving surgery	417	(31.5)	328	(37.2)	0.005	310	(35.1)	328	(37.2)	0.372
Mastectomy	908	(68.5)	554	(62.8)		572	(64.9)	554	(62.8)	
**Insurance**										
Uninsured	32	(2.4)	35	(4.0)	0.166	23	(2.6)	35	(4.0)	0.328
Insured	1012	(76.4)	669	(75.9)		667	(75.6)	669	(75.9)	
Medicaid	260	(19.6)	168	(19.0)		178	(20.2)	168	(19.0)	
Unknown	21	(1.6)	10	(1.1)		14	(1.6)	10	(1.1)	
**Marital status**										
Married	593	(44.8)	437	(49.5)	0.025	420	(47.6)	437	(49.5)	0.588
Others	650	(49.1)	408	(46.3)		418	(47.4)	408	(46.3)	
Unknown	82	(6.2)	37	(4.2)		44	(5.0)	37	(4.2)	

Abbreviations: PSM, propensity-score matching; HRc, hormone receptor; HER2, human epidermal growth factor receptor 2. *Pearson’s chi-square test.

Propensity-score matching (PSM) (optimal, 1:1) between the no-radiotherapy and radiotherapy groups was performed applying all variables (age, race, T and N categories, histology, grade, molecular subtype, surgery type, insurance, marriage, bone/lung/liver metastases, and multiplicity of metastatic sites). After PSM, the no-radiotherapy (n = 882) and radiotherapy (n = 882) groups showed no statistical difference for any variable (Table 1). Of the patients, 1112 (63.0%), 299 (17.0%), and 284 (16.1%) were associated with bone, lung, and liver metastases, indicating that more than 60% involved bone. There was no significant difference in surgery type among molecular subtypes (p = 0.457) (Supplementary Table S1).

### Comparison of 3-year cancer-specific survival and overall survival

Since the SEER database provided information of the metastatic site from 2010, the follow-up period for patients enrolled in the study had been short. Therefore, only 3-year cancer-specific survival (CSS) and overall survival (OS) rather than 5-year CSS and OS could be obtained. The 3-year CSS in the no-radiotherapy and radiotherapy groups after PSM were 57.1% and 70.9%, respectively (P < 0.001) (Table 2). In subgroup analysis, the radiotherapy group demonstrated consistently favorable CSS rates compared to the no-radiotherapy group, except for undifferentiated tumor grade (66.7% vs. 40.0%, P = 0.619) and hormone receptor-negative/human epidermal growth factor receptor 2 (HER2)-positive (67.8% vs. 80.3%, P = 0.360) patients. Patients with lung metastasis showed marginal benefit from radiotherapy, with the 3-year CSS increasing from 48.0% to 58.8% (P = 0.053) with the addition of radiotherapy. Radiotherapy increased the 3-year CSS for patients with liver metastasis (44.1% vs. 63.6%, P = 0.001) and visceral metastasis (lung and/or liver metastasis) (47.0% vs. 62.0%, P < 0.001). Patients with both lung and liver metastases showed a 2-year CSS of 38.0% and 56.0% in the no-radiotherapy and radiotherapy groups, respectively, but it was not statistically significant (P = 0.176). Radiotherapy increased the 3-year CSS for patients with multiple metastases (40.9% vs. 59.2%, P = 0.028).

**Table 2:** Comparison of CSS rates between the no-radiotherapy and radiotherapy groups in each subgroup after PSM in stage IV breast cancer.

Characteristics	Surgery only (n = 882)		Surgery plus radiotherapy (n = 882)		p*
	3-year CSS (%)	95% CI	3-year CSS (%)	95% CI	
All patients	57.1	(52.5–61.4)	70.9	(66.5–74.8)	<0.001
**Age**					
<50	59.7	(50.5–67.7)	70.5	(61.4–77.9)	0.013
50–59	58.3	(49.7–65.9)	75.7	(67.3–82.2)	0.001
60–69	55.7	(45.7–64.6)	69.3	(60.4–76.5)	0.010
≥70	53.8	(43.6–62.9)	65.7	(55.2–74.3)	0.030
**Race**					
White	59.5	(54.2–64.5)	71.8	(66.8–76.2)	<0.001
Black	40.1	(29.0–50.9)	63.4	(51.0–73.4)	<0.002
Others	71.5	(54.7–82.9)	74.2	(57.5–85.1)	0.738
Unknown	NA	NA		NA	NA
**T stage**					
≤T1	65.2	(52.4–75.4)	78.9	(66.5–87.2)	0.050
T2	65.2	(58.1–71.4)	75.3	(68.4–80.9)	0.003
T3	51.0	(39.7–61.1)	70.6	(59.4–79.3)	0.001
T4	44.5	(35.5–53.0)	63.9	(55.2–71.4)	<0.001
Unknown	67.9	(32.9–87.3)	NA	NA	0.382
**N stage**					
N0	59.5	(49.3–68.3)	72.0	(60.7–80.5)	0.034
N1	56.7	(48.5–64.1)	77.4	(69.9–83.2)	<0.001
N2	58.3	(47.9–67.3)	69.7	(59.4–77.9)	0.026
N3	51.2	(41.3–60.2)	66.3	(57.8–73.6)	0.012
Unknown	84.6	(51.2–95.9)	51.2	(23.1–73.7)	0.046
**Histology**					
Invasive ductal carcinoma	55.4	(49.9–60.5)	69.8	(64.7–74.3)	<0.001
Invasive lobular carcinoma	63.3	(48.2–75.1)	77.9	(63.2–87.3)	0.047
Others	57.5	(45.2–68.0)	74.4	(62.8–82.8)	0.162
Unknown	90.0	(47.3–98.5)	NA	NA	0.138
**Grade**					
Well differentiated	75.1	(54.3–87.5)	95.7	(83.8–98.8)	0.010
Moderately differentiated	67.8	(59.8–74.5)	80.6	(73.7–85.8)	<0.001
Poorly differentiated	47.5	(41.4–53.5)	62.9	(56.3–68.7)	<0.001
Undifferentiated	66.7	(19.5–90.4)	40.0	(9.7–69.8)	0.619
Unknown	70.8	(51.4–83.6)	53.9	(28.1–74.0)	0.618
**IHC subtype**					
HRc+/HER2-	63.2	(56.8–68.9)	75.0	(69.3–79.8)	<0.001
HRc+/HER2+	65.9	(51.6–76.9)	78.5	(67.0–86.4)	0.038
HRc−/HER2+	67.8	(51.9–79.5)	80.3	(66.0–89.1)	0.360
HRc−/HER2−	22.1	(12.9–32.9)	36.0	(24.0–48.1)	0.002
Unknown	56.3	(39.8–69.8)	76.8	(58.3–87.8)	0.034
**Bone metastasis**					
No	53.4	(46.1–60.2)	72.0	(64.9–78.0)	<0.001
Yes	58.9	(52.9–64.5)	70.2	(64.5–75.1)	0.003
**Lung metastasis**					
No	59.0	(53.9–63.7)	73.5	(68.8–77.7)	<0.001
Yes	48.0	(37.1–58.0)	58.8	(47.8–68.2)	0.053
**Liver metastasis**					
No	59.7	(54.7–64.4)	72.2	(67.5–76.4)	<0.001
Yes	44.1	(32.9–54.7)	63.6	(51.9–73.3)	0.001
**Combination of metastatic sites**					
Bone metastasis only	64.3	(57.5–70.4)	73.1	(66.6–78.6)	0.031
Lung metastasis only	52.1	(37.0–65.3)	58.6	(41.1–72.6)	0.163
Liver metastasis only	53.0	(36.9–66.7)	73.2	(57.3–84.0)	0.015
Other metastasis only	55.1	(45.1–64.1)	78.2	(68.8–85.0)	<0.001
Bone and lung metastases	44.2	(27.8–59.4)	61.6	(47.3–73.0)	0.196
Bone and liver metastases	35.9	(19.9–52.2)	57.5	(39.1–72.1)	0.018
Lung and liver metastases^†^	38.0^†^	(14.9–61.1)	56.0^†^	(28.1–76.7)	0.176
Lung and/or liver metastases	47.0	(38.9–54.6)	62.0	(53.7–69.3)	<0.001
**Multiple sites of metastases**					
No	60.0	(55.0–64.6)	73.0	(68.3–77.1)	<0.001
Yes	40.9	(28.9–52.5)	59.2	(47.6–69.0)	0.028
**Surgery**					
Breast-conserving surgery	59.0	(51.2–66.0)	73.6	(66.2–79.6)	<0.001
Mastectomy	56.2	(50.4–61.5)	69.2	(63.6–74.1)	<0.001
**Insurance**					
Uninsured	38.4	(14.2–62.4)	58.7	(26.1–80.9)	0.036
Insured	58.7	(53.4–63.6)	71.3	(66.4–75.6)	<0.001
Medicaid	53.3	(42.7–62.9)	71.2	(59.8–79.9)	0.010
Unknown	47.6	(8.18–80.3)	67.5	(29.1–88.3)	0.714
**Marital status**					
Married	58.8	(52.0–65.0)	73.0	(66.5–78.4)	<0.001
Others	54.4	(47.6–60.6)	67.6	(61.3–73.3)	<0.001
Unknown	66.5	(44.5–81.5)	79.1	(54.4–91.3)	0.438

Abbreviations: CSS, cancer-specific survival; PSM, propensity-score matching; CI, confidence interval; NA, not applicable; HRc, hormone receptor; HER2, human epidermal growth factor receptor 2. *Kaplan-Meier survival estimate compared by a log-rank test. ^†^Two-year CSS.

The 3-year OS in the no-radiotherapy and radiotherapy groups were 53.6% and 68.4%, respectively (P < 0.001). The results of a comparison of OS rates between the no-radiotherapy and radiotherapy groups were similar to those for CSS (Supplementary Table S2).

### Univariate and multivariate analyses for 3-year CSS and OS

On univariate analysis for CSS, old age (≥70), black race, high T category, high grade, hormone receptor-/HER2- molecular subtype, lung metastasis, liver metastasis, and multiple sites of metastasis were poor prognostic factors, while radiotherapy and married status were favorable prognostic factors (Table 3).

**Table 3:** Univariate and multivariate analyses for CSS rate after PSM in stage IV breast cancer.

Characteristics	Univariate			Multivariate		
	3-year CSS (%)	95% CI	p*	HR	95% CI	*p* ^†^
**Age**						
<50	65.1	(58.8–70.8)		Reference		
50–59	66.8	(60.8–72.1)	0.006	1.064	(0.816–1.386)	0.647
60–69	63.0	(56.5–68.8)		1.213	(0.926–1.590)	0.161
≥70	59.6	(52.4–66.0)		1.709	(1.283–2.276)	<0.001
**Race**						
White	65.8	(62.2–69.2)		Reference		
Black	51.0	(42.7–58.7)	<0.001	1.060	(0.827–1.359)	0.644
Others	73.2	(62.1–81.5)		0.768	(0.507–1.165)	0.214
Unknown	NA	NA		NA	NA	NA
**T stage**						
≤T1	71.9	(63.2–78.9)		Reference		
T2	70.2	(65.3–74.5)	<0.001	1.054	(0.757–1.468)	0.755
T3	60.7	(52.9–67.8)		1.196	(0.826–1.732)	0.344
T4	54.8	(48.5–60.7)		1.379	(0.984–1.931)	0.062
Unknown	57.8	(35.3–74.9)		1.459	(0.739–2.880)	0.276
**N stage**						
N0	65.0	(57.6–71.4)		Reference		
N1	67.1	(61.6–72.0)	0.125	0.822	(0.618–1.093)	0.177
N2	34.2	(57.1–70.5)		0.939	(0.688–1.284)	0.695
N3	59.7	(53.2–65.5)		1.184	(0.881–1.591)	0.263
Unknown	60.0	(36.4–77.3)		0.904	(0.456–1.791)	0.771
**Histology**						
Invasive ductal carcinoma	62.8	(59.1–66.3)				
Invasive lobular carcinoma	70.4	(60.3–78.4)	0.576	NA		
Others	65.5	(57.2–72.7)				
Unknown	56.1	(15.0–83.8)				
**Grade**						
Well differentiated	86.5	(75.7–92.7)		Reference		
Moderately differentiated	74.6	(69.5–78.9)	<0.001	1.349	(0.781–2.332)	0.283
Poorly differentiated	55.0	(50.5–59.3)		2.322	(1.360–3.964)	0.002
Undifferentiated	43.8	(15.1–69.7)		3.436	(1.435–8.228)	0.006
Unknown	63.1	(46.7–75.7)		2.305	(1.192–4.456)	0.013
**IHC subtype**						
HRc+/HER2−	69.4	(65.2–73.2)		Reference		
HRc+/HER2+	72.8	(64.1–79.7)	<0.001	0.685	(0.494–0.952)	0.024
HRc−/HER2+	73.4	(62.7–81.5)		0.737	(0.490–1.108)	0.142
HRc−/HER2−	28.3	(20.6–36.4)		2.857	(2.228–3.664)	<0.001
Unknown	65.1	(52.8–74.9)		1.393	(0.948–2.046)	0.091
**Bone metastasis**						
No	63.0	(58.0–67.7)				
Yes	64.6	(60.5–68.4)	0.336	NA		
**Lung metastasis**						
No	66.3	(62.9–69.5)		Reference		
Yes	53.5	(45.8–60.6)	<0.001	1.117	(0.847–1.473)	0.434
**Liver metastasis**						
No	66.1	(62.7–69.3)		Reference		
Yes	53.7	(45.6–61.1)	<0.001	1.489	(1.105–2.006)	0.009
**Multiple sites of metastases**						
No	66.6	(63.2–69.8)		Reference		
Yes	50.1	(41.7–57.9)	<0.001	1.555	(1.126–2.146)	0.007
**Surgery**						
Breast-conserving surgery	66.6	(61.4–71.4)				
Mastectomy	62.6	(58.6–66.3)	0.255	NA		
**Radiotherapy**						
No	57.1	(52.5–61.4)		Reference		
Yes	70.9	(66.5–74.8)	<0.001	0.572	(0.472–0.693)	<0.001
**Insurance**						
Uninsured	52.4	(31.3–69.9)		Reference		
Insured	65.1	(61.6–68.5)	0.091	0.603	(0.365–0.996)	0.048
Medicaid	61.9	(54.2–68.7)		0.562	(0.329–0.961)	0.035
Unknown	54.7	(22.0–78.6)		1.004	(0.410–2.460)	0.993
**Marital status**						
Married	66.1	(61.5–70.3)		Reference		
Others	61.1	(56.5–65.4)	0.008	1.198	(0.973–1.475)	0.089
Unknown	72.0	(56.3–82.9)		0.837	(0.499–1.405)	0.502

Abbreviations: CSS, cancer-specific survival; PSM, propensity-score matching; CI, confidence interval; HR, hazard ratio; NA, not applicable; HRc, hormone receptor; HER2, human epidermal growth factor receptor 2. ^*^Kaplan-Meier survival estimate compared by a log-rank test. ^†^Cox proportional hazards model.

**Figure 1:**
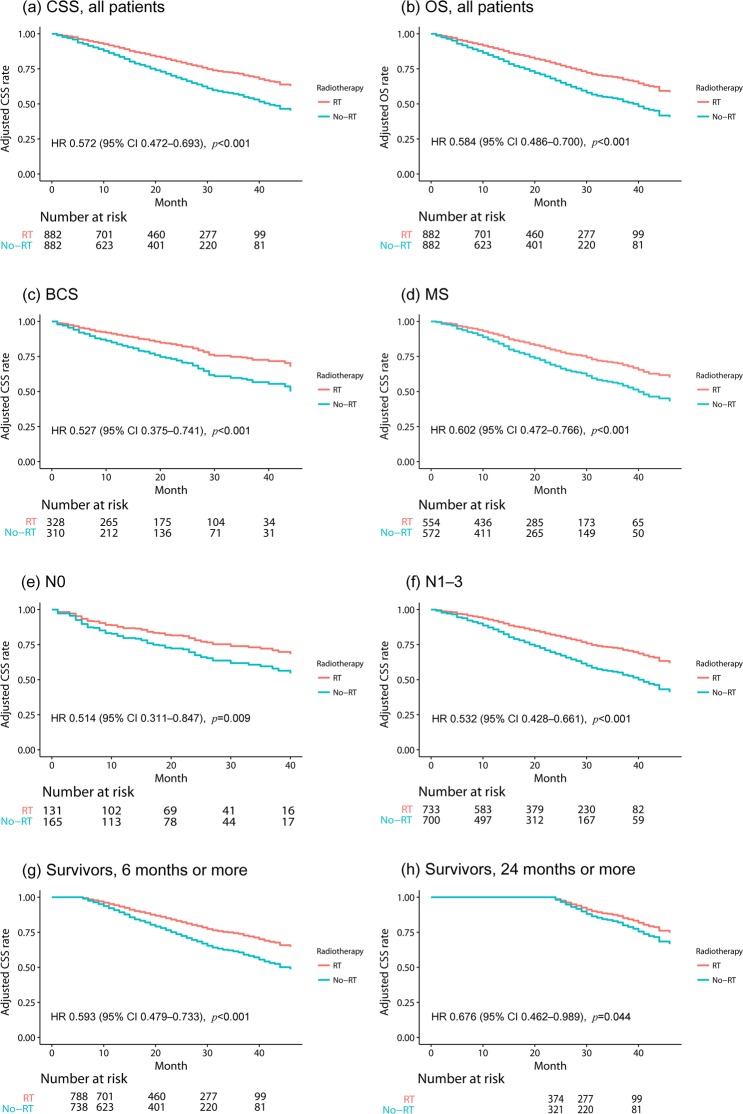
Comparison of CSS and OS between the no-radiotherapy and radiotherapy groups shows significantly favorable CSS and OS in the radiotherapy group. All survival curves were adjusted using the Cox proportional hazard model. (**a**) CSS in all patients, (**b**) OS in all patients, (**c**) CSS in patients who underwent breast-conserving surgery, (**d**) CSS in patients who underwent mastectomy, (**e**) CSS in patients without lymph node involvement (N0), (**f**) CSS in patients with lymph node involvement (N1–3), (**g**) CSS in patients who survived 6 months or more from the time of diagnosis, and (**h**) CSS in patients who survived 24 months or more from the time of diagnosis. CSS, cancer-specific survival; OS, overall survival; BCS, breast-conserving surgery; MS, mastectomy; RT, radiotherapy; HR, hazard ratio; CI, confidence interval.

After incorporating variables with two-tailed P values < 0.2 in univariate analysis, multivariate analysis for CSS was performed. Old age (≥70), high grade, hormone receptor-/HER2- subtype, liver metastasis, and multiple metastases remained as adverse prognostic factors, and radiotherapy was a significantly favorable factor (hazard ratio [HR], 0.572; 95% confidence interval [CI], 0.472–0.693; P < 0.001) (Table 3) (Fig. 1a).

Univariate and multivariate analyses results for OS were similar to those for CSS. Radiotherapy remained a significantly favorable factor for OS after multivariate analysis (HR, 0.584; 95% CI, 0.486–0.700; P < 0.001) (Supplementary Table S3) (Fig. 1b).

### Subgroup analysis according to surgery type

As radiotherapy after surgery might be correlated with surgery type, patients were divided according to surgery type (breast-conserving surgery or mastectomy), and univariate and multivariate analyses were performed for each surgery group. There were 638 patients (310 vs. 328 in the no-radiotherapy and radiotherapy groups) in the breast-conserving surgery group and 1126 (572 vs. 554 in the no-radiotherapy and radiotherapy groups) in the mastectomy group.

In the breast-conserving surgery group, multivariate analysis for CSS incorporating variables with two-tailed P values < 0.2 in univariate analysis demonstrated that radiotherapy was significantly correlated with a favorable CSS (HR, 0.527; 95% CI, 0.375–0.741; P < 0.001) (Fig. 1c). Radiotherapy was also a significantly favorable prognostic factor in the mastectomy group (HR, 0.602; 95% CI, 0.472–0.766; P < 0.001) (Fig. 1d).

Multivariate analyses for OS in both surgery groups showed similar results (HR of radiotherapy in the breast-conserving surgery group, 0.535; 95% CI, 0.387–0.740; P < 0.001; HR of radiotherapy in the mastectomy group, 0.624; 95% CI, 0.497–0.783; P < 0.001) (Supplementary Fig. S1).

### Subgroup analysis according to N stage

Not only may the type of surgery, but also lymph node metastasis determine the application of radiotherapy after surgery. Therefore, we performed subgroup analyses according to the lymph node involvement. The number of patients with and without lymph node metastasis was 296 (165 vs. 131 in the no-radiotherapy and radiotherapy groups) and 1433 (700 vs. 733 in the no-radiotherapy and radiotherapy groups), respectively. Multivariate analysis incorporating variables with two-tailed P values < 0.2 in each univariate analysis showed favorable CSS after radiotherapy in patients with lymph node involvement (HR 0.532, 95% CI 0.428–0.661, P < 0.001) (Fig. 1f) and even in patients without lymph node involvement (HR 0.514, 95% CI 0.311–0.847, P = 0.009) (Fig. 1e). Radiotherapy also improved the OS irrespective of lymph node involvement (in patients with lymph node involvement, HR 0.541, 95% CI 0.441–0.665, P < 0.001; in patients without lymph node involvement, HR 0.564, 95% CI 0.352–0.901, P = 0.017) (Supplementary Fig. S1).

### Subgroup analysis for patients who survived ≥6 months and ≥24 months

Patients with short life expectancies because of their comorbidities or poor performance status may undergo radiotherapy less often after surgery than those who have a longer life expectancy. To reduce the selection bias for radiotherapy related to patients’ general condition, patients who survived ≥6 months and ≥24 months were selected, and univariate and multivariate analyses for CSS and OS were performed. No significant difference was observed between the no-radiotherapy and radiotherapy groups (Supplementary Table S4). There were 738 and 788 patients in the no-radiotherapy and radiotherapy groups, respectively, who survived ≥6 months. Multivariate analyses revealed that radiotherapy still improved CSS (HR, 0.593; 95% CI, 0.479–0.733; P < 0.001) and OS (HR, 0.608; 95% CI, 0.497–0.745; P < 0.001) (Fig. 1g and Supplementary Fig. S1).

There were 321 and 374 in the no-radiotherapy and radiotherapy groups, respectively, who survived ≥24 months. On multivariate analyses for CSS and OS, radiotherapy still provided significantly favorable prognoses (HR for CSS, 0.676; 95% CI, 0.462–0.989, P = 0.044; HR for OS, 0.678; 95% CI, 0.476–0.964; P = 0.030) (Fig. 1h and Supplementary Fig. S1).

## Discussion

Radiotherapy after surgery in *de novo* stage IV breast cancer significantly improved CSS and OS rates compared with surgery alone. Even for patients with visceral or multiple metastases, radiotherapy showed a survival benefit. This tendency of the survival benefit of radiotherapy after surgery was observed irrespective of surgery type and lymph node involvement and maintained in patients who survived ≥6 months and ≥24 months after diagnosis.

Badwe *et al*.^19^ demonstrated that surgery and radiotherapy in *de novo* stage IV breast cancer did not increase survival. In this prospective randomized trial, patients who underwent surgery and radiotherapy showed a remarkably favorable loco-regional progression-free survival compared with the no local treatment group. However, the local treatment group had a significantly worse distant progression-free survival, resulting in no difference in OS between the two groups. This was correlated to the treatment-provoked growth of a metastatic tumor after local treatments to the primary site^27–29^. In this study, patients were treated with systemic chemotherapy before randomization, and only responsive patients were enrolled. After local treatments or observation, further systemic therapy was not administered until disease progression in both groups, suggesting that the accelerated growth of the metastatic tumors triggered by local treatments was not controlled with additional systemic therapy. Moreover, this study excluded patients with a single metastasis amenable to local treatments with curative intent, who were expected to have a favorable prognosis.

Another prospective registry study^20^ also demonstrated no survival benefit of surgery and radiotherapy for patients with *de novo* stage IV breast cancer; even patients with a single metastasis did not achieve a survival benefit. The treatment setting, however, is similar to that of Badwe *et al*.^19^, wherein patients were treated with systemic therapy before local treatment but not after local treatment. Additionally, the follow-up was relatively short; only 3-year OS could be obtained from this study.

In contrast, in a recent randomized controlled trial^17^ local treatments (surgery and radiotherapy) showed a 5-year survival benefit. In this study, patients in the local treatment group underwent surgery and radiotherapy as the first treatment, followed by chemotherapy. The observation group received chemotherapy immediately after randomization. The survival benefit of local treatments did not appear until 3 years after randomization^30^. However, after 5 years, the local treatment group showed significantly improved survival^17^. Several population-based retrospective studies have also demonstrated survival benefit of local treatments in *de novo* stage IV breast cancer^13,14,21,22^.

One of our most important findings is the role of radiotherapy for increasing survival in stage IV breast cancer. In our study, radiotherapy after surgery showed a significant survival benefit after PSM, regardless of surgery type, lymph node involvement, and the burden of distant metastasis, and even after examining only patients who survived 6 or 24 months or more. This suggests that radiotherapy combined with surgery during the diagnostic period might have independent therapeutic value to improve survival.

In our study, the no-radiotherapy group patients are also likely to undergo palliative radiotherapy at some point during their life span. Therefore, our study might suggest that the interval time between surgery and radiotherapy affects survival in metastatic breast cancer. Generally, the local recurrence rate increases with an increase in the waiting time for radiotherapy^31^. In stage IV breast cancer, the relationship between survival rate and time interval between surgery and radiotherapy is scarcely studied. Clinical guidelines recommend both local treatment and systemic therapy for stage IV breast cancer patients, and the order and timing of treatments are left to the clinician’s judgment^6^. Clinicians determine the treatment based on the molecular subtype of breast cancers, the applicability of effective systemic therapies, and the symptoms of the metastatic region^32^.

However, it might no longer be reasonable to consider radiotherapy as merely palliative therapy, delaying it until the response to systemic therapy is confirmed, or until palliative therapy is inevitable. If radiotherapy is performed at the time of the diagnosis of metastatic cancer, tumor burden can be reduced before initiating systemic therapy, and an abscopal effect may be possible by administering radiotherapy with immune therapy^33^. Therefore, radiotherapy may play a more important role than conventionally considered in metastatic breast cancer^34,35^.

Radiotherapy is an effective and universal treatment for bone metastasis among several metastatic lesions^36^. In our study, 63.0% of patients had bone metastasis, and 48.8% of patients had only bone metastasis. Therefore, many patients in our study may have undergone radiotherapy for bone metastasis. Recently, one randomized controlled study demonstrated that stereotactic ablative radiotherapy (SABR) improved survival rate in patients with oligo-metastasis compared to conventional palliative radiotherapy^35^. Other studies have reported that hypofractionated stereotactic radiotherapy improves survival, especially in bone metastasis^34^. Therefore, radiotherapy in stage IV patients with low tumor burden, such as bone metastasis, may improve survival rate by improving the local control rate of metastatic lesions.

The inability to identify the specific site where radiotherapy was administered is a major weakness of our study. The effect of radiotherapy on survival rate in our study may vary depending on the location and number of treatment sites. Therefore, our study cannot conclude which site of radiotherapy (tumor bed or metastatic regions except brain) may help to improve survival. Our study only suggests that active local treatment by not only surgery but also radiotherapy need to be considered as the primary treatment in *de novo* stage IV breast cancer, and further studies are needed to identify the site and regimen (adjuvant radiotherapy to the tumor bed, conventional palliative radiotherapy to the metastatic sites, or stereotactic ablative radiotherapy to metastatic sites expecting to induce abscopal effect, etc.) of radiotherapy that may improve survival.

Without information on performance status and chemotherapy, selection bias may still exist even after PSM. In our study, however, patients with brain metastasis, whose performance would deteriorate rapidly, were excluded, and patients were analyzed separately according to the severity of distant metastases. Moreover, patients who survived 6 or 24 months or more still had a survival benefit from radiotherapy, suggesting that the survival benefit from radiotherapy might not be solely explained by the selection bias, even though the administration of radiotherapy may be correlated with performance status at diagnosis. All patients underwent surgery, indicating that the difference in comorbidities between the no-radiotherapy and radiotherapy groups might be minor. When we studied, the SEER database did not provide data on chemotherapy administration. Patients treated with radiotherapy might be treated with chemotherapy more actively than those who were not. Therefore, the radiotherapy group might show better survival than that shown by the no-radiotherapy group. However, it is hard to speculate that stage IV breast cancer patients who had a reasonable performance status for surgery might not be treated with chemotherapy.

In conclusion, notwithstanding the limitations of this study, our study showed that radiotherapy after surgery has a possibility to increase survival in *de novo* stage IV breast cancer by using the one of the largest, most recently diagnosed, and propensity-score matched patient population data. The significant survival benefits from radiotherapy in *de novo* stage IV breast cancer patients who were diagnosed recently (2010–2013) when advanced systemic therapies are available, suggests that active treatment using radiotherapy after surgery may improve the survival rate for patients with *de novo* stage IV breast cancer.

## Conclusions

In *de novo* stage IV breast cancer, surgery with radiotherapy improved the CSS and OS rates compared to surgery without radiotherapy, suggesting that active treatments using radiotherapy may improve survival in *de novo* stage IV breast cancer, especially with synchronous bone metastasis.

## Methods

### Study population

Population data were obtained from the SEER database 18 (2010–2013) after agreeing to the terms and conditions of data use. The data is available at https://seer.cancer.gov/seertrack/data/request/. Ethical approval and informed consent to participate was waived by the Ewha Womans University Institutional Review Board, since de-identified data from the SEER registry was used for this study. The investigators were not involved in the process of data collection or entry.

The definition of *de novo* stage IV cancer is stage IV breast cancer at first diagnosis, and therefore, a *de novo* stage IV cancer patient is a patient who has not undergone cancer treatment before the stage IV cancer diagnosis.

Inclusion criteria were as follows: female; aged 19 or older; being diagnosed with M1 stage breast cancer at the first diagnosis (*de novo* stage IV) between 2010 and 2013; having information on metastases in the bone, liver, and lung; surgery to the primary sites (breast-conserving surgery code: 20–24, mastectomy code: 40–41, 43–46, 50–51, 53–56, 60–61, 64–67, 70–71); and having information on radiotherapy administration (postoperative external beam radiotherapy or no radiotherapy). The SEER database does not provide information about the site of radiotherapy. Therefore, the site of the surgery was the primary site (breast) and the site of the radiotherapy after the surgery could be the primary site and/or metastatic sites.

The SEER database offers only the first course treatment information at the time of diagnosis and does not provide treatment information after relapse or progression. Therefore, all treatments in this study were the first course treatment after being diagnosed with *de novo* stage IV breast cancer. Patients diagnosed with brain metastasis or other cancer prior to breast cancer and those treated by removal of the uninvolved contralateral breast were excluded. Patients who survived less than 1 month were also excluded.

Information on tumor grade (4 points) has been provided since the beginning of SEER registration, and the Bloom-Richardson (BR) grade (3 points) has been available since 2004. Well, moderate, and poorly differentiated tumor grades are almost identical to BR grades 1, 2, and 3, respectively, and undifferentiated tumor grade is mainly included in BR grade 3 or unknown BR grade. Prior to PSM, the prevalences of unknown tumor grade and unknown BR grade were 6.6% (145/2207) and 13.7% (303/2207), respectively. Therefore, we performed analyses using the tumor grade rather than the BR grade.

### Statistical analysis

Patients were divided into groups based on local treatment: surgery alone (no-radiotherapy group) and surgery followed by radiotherapy (radiotherapy group). Comparisons of patient characteristics were assessed by Pearson’s chi-square test. One-to-one (1:1) PSM was performed to construct a matched sample consisting of pairs of no-radiotherapy and radiotherapy subjects using an optimal matching algorithm. Variables that were significantly different between the two groups by Pearson’s chi-square test or considered to be clinically important were used to generate propensity scores.

After PSM, the 3-year CSS and OS were calculated using the Kaplan-Meier method. CSS was defined as the time duration between the date of diagnosis to death owing to breast cancer, and was censored for the last follow-up date for patients who were alive or dead due to other causes. The SEER program provides information on the cause of death (“dead attributable to this cancer diagnosis” or “alive or dead of other cause”). OS was defined as the time interval from the date of diagnosis to the date of death due to any cause, and was censored for the last follow-up date for patients who were alive. CSS and OS rates between the no-radiotherapy and radiotherapy groups were compared using a log-rank test. Variables with two-tailed P values < 0.2 were incorporated in a multivariate analysis. The multivariate analysis was performed using Cox proportional hazard model.

A two-tailed P value < 0.05 was considered statistically significant. PSM was performed using R software ver. 3.3.3 for Windows (http://cran.r-project.org) with the R packages ‘MatchIt’^37^ and ‘opmatch’^38^. Adjusted survival curves for the Cox proportional hazards model were generated using ‘survminer’^39^ package. Other statistical analyses were performed using Stata MP ver. 14.2 for Windows (Stata Corporation, College Station, TX, USA).

## Supplementary information

Supplementary tables and figure

## Data Availability

The raw datasets of the current study are available in the SEER repository, https://seer.cancer.gov/seertrack/data/request/.
