# Supplementary material for: Survival benefit of radiotherapy after surgery in de novo stage IV breast cancer: a population-based propensity-score matched analysis
Source: Sci Rep. 2019 Jun 12;9:8527. doi: 10.1038/s41598-019-45016-2 (PMC6561914; doi:10.1038/s41598-019-45016-2)

Survival benefit of radiotherapy after surgery in de novo stage IV breast cancer: a population-based propensity-score matched analysis

Yi-Jun Kim, So-Youn Jung, Kyubo Kim

Table S1. Comparison of surgery type according to molecular subtype after PSM in stage IV breast cancer

Characteristics	HR+/HER2- n=965		HR+/HER2+ n=290		HR-/HER2+ n=140		HR-/HER2- n=277		Unknown n=92		*p**
	no.	(%)	no.	(%)	no.	(%)	no.	(%)	no.	(%)	
Surgery											
Breast-conserving surgery	358	(37.1)	103	(35.5)	41	(29.3)	100	(36.1)	36	(39.1)	0.457
Mastectomy	607	(62.9)	187	(64.5)	99	(70.7)	177	(63.9)	56	(60.9)	

Abbreviations: PSM, propensity score matching; HR, hormone receptor; HER2, human epidermal growth factor receptor 2. *Pearson's chi-square test.

Table S2. Comparison of OS rates between the no-radiotherapy and radiotherapy groups in each subgroup after PSM in stage IV breast cancer

Characteristics	Surgery only (n=882)		Surgery and radiotherapy (n=882)		p†
	3-year OS (%)	95% CI	3-year OS (%)	95% CI	
All patients	53.6	(49.1–58.0)	68.4	(64.1–72.4)	<0.001
Age					
<50	57.3	(48.2–65.4)	69.7	(60.7–77.1)	0.006
50–59	55.8	(47.3–63.6)	73.9	(65.4–80.6)	<0.001
60–69	54.7	(44.8–63.5)	67.8	(59.0–75.1)	0.020
≥70	44.6	(35.2–53.7)	58.4	(48.1–67.3)	0.017
Race					
White	56.0	(50.7–60.9)	69.6	(64.5–74.0)	<0.001
Black	35.9	(25.3–46.5)	60.8	(48.8–70.8)	0.001
Others	71.5	(54.7–82.9)	70.3	(53.8–81.8)	0.983
Unknown	NA	NA	NA	NA	NA
T stage					
≤T1	61.7	(49.1–72.0)	77.0	(64.6–85.5)	0.040
T2	61.0	(53.7–67.0)	72.6	(65.7–78.3)	0.004
T3	49.5	(38.5–59.5)	69.5	(58.4–78.3)	<0.001
T4	40.6	(32.0–49.1)	61.1	(52.4–68.7)	<0.001
Unknown	67.9	(32.9–87.3)	43.0	(16.1–67.7)	0.265
N stage					
N0	57.3	(47.2–66.2)	69.8	(58.6–78.5)	0.046
N1	51.9	(43.9–59.4)	75.0	(67.5–81.0)	<0.001
N2	56.2	(46.0–65.3)	68.1	(57.8–76.4)	0.051
N3	48.0	(38.4–57.1)	62.6	(54.1–70.0)	0.010
Unknown	74.7	(45.5–89.7)	25.6	(1.8–63.2)	0.100
Histology					
Invasive ductal carcinoma	52.3	(46.9–57.4)	67.2	(62.1–71.8)	<0.001
Invasive lobular carcinoma	61.2	(46.5–73.1)	76.8	(62.2–86.4)	0.043
Others	51.9	(39.8–62.6)	71.3	(59.4–80.3)	0.069
Unknown	82.5	(46.1–95.3)	NA	NA	0.371
Grade					
Well differentiated	72.0	(51.3–85.1)	95.7	(83.8–98.9)	0.005
Moderately differentiated	65.3	(57.4–72.0)	77.9	(71.0–83.3)	0.001
Poorly differentiated	44.0	(38.0–49.9)	60.3	(53.9–66.2)	<0.001
Undifferentiated	66.7	(19.5–90.4)	40.0	(9.7–69.8)	0.619
Unknown	64.3	(45.7–77.9)	50.5	(26.1–70.7)	0.978
IHC subtype					
HR+/HER2-	59.3	(53.0–65.1)	72.2	(66.4–77.1)	0.001
HR+/HER2+	64.1	(50.2–75.0)	75.3	(63.9–83.5)	0.040
HR-/HER2+	60.8	(45.0–73.3)	80.3	(66.0–89.1)	0.125
HR-/HER2-	21.1	(12.3–31.5)	35.6	(23.8–47.7)	<0.001
Unknown	53.7	(37.4–67.5)	72.7	(53.3–85.1)	0.043
Bone metastasis					
No	51.0	(43.8–57.7)	68.9	(61.7–75.0)	<0.001
Yes	54.9	(48.9–60.4)	68.1	(62.5–73.1)	0.001
Lung metastasis					
No	55.7	(50.7–60.5)	70.6	(65.8–74.9)	<0.001
Yes	43.5	(32.9–53.6)	58.3	(47.3–67.7)	0.021
Liver metastasis					
No	55.9	(50.9–60.5)	69.5	(64.8–73.8)	<0.001
Yes	42.5	(31.4–53.1)	62.3	(50.7–71.9)	0.002
Combination of metastatic sites					
Bone metastasis only	60.7	(53.9–66.8)	70.9	(64.4–76.5)	0.018
Lung metastasis only	50.6	(35.9–63.6)	58.6	(41.1–72.6)	0.095
Liver metastasis only	52.2	(36.4–65.8)	73.2	(57.3–84.0)	0.017
Other metastasis only	51.7	(41.9–60.6)	72.6	(63.2–80.0)	<0.001
Bone and lung metastases	37.2	(22.1–52.2)	60.4	(46.3–71.9)	0.113
Bone and liver metastases	33.5	(18.2–49.6)	55.1	(37.4–69.6)	0.028
Lung and liver metastases*	38.0*	(14.9–61.1)	43.7*	(18.4–66.7)	0.259
Lung and/or liver metastases	43.8	(35.8–51.4)	61.4	(53.1–68.6)	<0.001
Multiple sites of metastases					
No	56.9	(52.0–61.5)	70.3	(65.6–74.5)	<0.001
Yes	35.9	(24.6–47.3)	57.9	(46.5–67.7)	0.016
Surgery					
Breast conserving surgery	55.0	(47.2–62.1)	72.6	(65.3–78.7)	<0.001
Mastectomy	53.0	(47.3–58.3)	65.9	(60.3–70.9)	<0.001
Insurance					
Uninsured	38.4	(14.2–62.4)	58.7	(26.1–80.9)	0.036
Insured	55.2	(50.0–60.1)	69.0	(64.1–73.4)	<0.001
Medicaid	49.9	(39.4–59.6)	67.4	(56.1–76.5)	0.024
Unknown	43.3	(8.1–75.7)	67.5	(29.1–88.3)	0.940
Marital status					
Married	57.7	(50.9–63.9)	71.4	(64.9–76.9)	<0.001
Others	48.1	(41.6–54.4)	64.2	(57.8–69.9)	<0.001
Unknown	66.5	(44.5–81.5)	79.1	(54.4–91.3)	0.590

Abbreviations: OS, overall survival; PSM, propensity score matching; CI, confidence interval; NA, not applicable; HR, hormone receptor; HER2, human epidermal growth factor receptor 2. †Kaplan-Meier survival estimate compared by a log-rank test. *Two-year OS.

Table S3. Univariate and multivariate analyses for overall survival (OS) rate after PSM in stage IV breast cancer

Characteristics	Univariate			Multivariate		
	3-year OS (%)	95% CI	*p*†	HR	95% CI	*p*‡
Age						
<50	63.6	(57.2–69.2)		Reference		
50–59	64.7	(58.6–70.0)	<0.001	1.073	(0.831–1.385)	0.588
60–69	61.7	(55.3–67.6)		1.209	(0.931–1.571)	0.154
≥70	51.2	(44.3–57.7)		1.978	(1.515–2.582)	<0.001
Race						
White	62.9	(59.3–66.3)		Reference		
Black	47.5	(39.5–55.1)	<0.001	1.110	(0.879–1.403)	0.381
Others	71.0	(59.9–79.6)		0.750	(0.503–1.118)	0.158
Unknown	NA	NA		NA	NA	NA
T stage						
≤T1	69.1	(60.4–76.2)		Reference		
T2	66.8	(61.8–71.2)	<0.001	1.090	(0.799–1.489)	0.586
T3	59.4	(51.6–66.3)		1.156	(0.812–1.644)	0.422
T4	51.5	(45.3–57.3)		1.334	(0.969–1.836)	0.077
Unknown	54.8	(33.0–72.1)		1.364	(0.713–2.610)	0.349
N stage						
N0	62.8	(55.5–69.3)		Reference		
N1	63.5	(58.0–68.4)	0.111	0.893	(0.680–1.173)	0.418
N2	62.4	(55.2–68.7)		0.983	(0.728–1.327)	0.912
N3	56.2	(49.8–62.1)		1.241	(0.935–1.647)	0.135
Unknown	56.6	(34.3–73.9)		1.145	(0.634–2.068)	0.653
Histology						
Invasive ductal carcinoma	60.0	(56.3–63.5)				
Invasive lobular carcinoma	68.7	(58.7–76.8)	0.501	NA		
Others	61.1	(52.6–68.5)				
Unknown	53.5	(15.1–81.4)				
Grade						
Well differentiated	85.0	(73.9–91.6)		Reference		
Moderately differentiated	72.0	(66.9–76.4)	<0.001	1.479	(0.87.–2.505)	0.145
Poorly differentiated	51.8	(47.4–56.1)		2.487	(1.484–4.170)	0.001
Undifferentiated	43.8	(15.1–69.7)		3.300	(1.393–7.820)	0.007
Unknown	58.1	(42.5–70.9)		2.689	(1.442–5.016)	0.002
IHC subtype						
HR+/HER2-	66.0	(61.8–69.9)		Reference		
HR+/HER2+	70.3	(61.8–77.3)	<0.001	0.722	(0.533–0.979)	0.036
HR-/HER2+	69.3	(58.4–77.9)		0.750	(0.512–1.100)	0.141
HR-/HER2-	27.5	(20.0–35.5)		2.686	(2.115–3.410)	<0.001
Unknown	61.9	(49.5–72.2)		1.296	(0.895–1.878)	0.170
Bone metastasis						
No	60.2	(55.1–64.8)				
Yes	61.5	(57.5–65.4)	0.373	NA		
Lung metastasis						
No	63.2	(59.8–66.5)		Reference		
Yes	51.0	(43.4–58.1)	<0.001	1.072	(0.820–1.401)	0.610
Liver metastasis						
No	62.8	(59.4–66.0)		Reference		
Yes	52.3	(44.3–59.7)	<0.001	1.384	(1.038–1.845)	0.027
Multiple sites of metastases						
No	63.7	(60.3–66.9)		Reference		
Yes	46.8	(38.6–54.6)	<0.001	1.590	(1.167–2.168)	0.003
Surgery						
Breast conserving surgery	64.2	(58.9–69.0)				
Mastectomy	59.4	(55.4–63.1)	0.236	NA		
Radiotherapy						
No	53.6	(49.1–58.0)		Reference		
Yes	68.4	(64.1–72.4)	<0.001	0.584	(0.486–0.700)	<0.001
Insurance						
Uninsured	52.4	(31.3–69.9)		Reference		
Insured	62.2	(58.7–65.5)	0.161	0.628	(0.381–1.035)	0.068
Medicaid	58.3	(50.6–65.2)		0.589	(0.347–1.000)	0.050
Unknown	51.5	(21.0–75.4)		1.085	(0.462–2.552)	0.851
Marital status						
Married	64.7	(60.1–69.0)		Reference		
Others	56.2	(51.7–60.5)	<0.001	1.288	(1.057–1.570)	0.012
Unknown	72.0	(56.3–82.9)		0.830	(0.502–1.371)	0.466

Abbreviations: OS, overall survival; PSM, propensity score matching; CI, confidence interval; HR, hazard ratio; NA, not applicable; HR, hormone receptor; HER2, human epidermal growth factor receptor 2. †Kaplan-Meier survival estimate compared by a log-rank test. ‡Cox proportional hazards model.

Table S4. Characteristics of stage IV breast cancer patients who survived ≥6 months and ≥24 months after PSM

Characteristics	Survived ≥6 months					Survived ≥24 months				
	Surgery only n=738		Surgery and radiotherapy n=788		*p**	Surgery only n=321		Surgery and radiotherapy n=374		*p**
	no.	(%)	no.	(%)		no.	(%)	no.	(%)	
Age										
<50	204	(27.6)	220	(27.9)		94	(29.3)	106	(28.3)	
50–59	212	(28.7)	240	(30.5)	0.479	104	(32.4)	108	(28.9)	0.549
60–69	185	(25.1)	205	(26.0)		67	(20.9)	94	(25.1)	
≥70	137	(18.6)	123	(15.6)		56	(17.4)	66	(17.6)	
Race										
White	558	(75.6)	599	(76.0)		246	(76.6)	294	(78.6)	
Black	127	(17.2)	116	(14.7)	0.152	46	(14.3)	46	(12.3)	0.684
Others	50	(6.8)	72	(9.1)		29	(9.0)	33	(8.8)	
Unknown	3	(0.4)	1	(0.1)		0	(0.0)	1	(0.3)	
T stage										
≤T1	102	(13.8)	99	(12.6)		51	(15.9)	55	(14.7)	
T2	286	(38.8)	301	(38.2)	0.670	140	(43.6)	140	(37.4)	0.349
T3	138	(18.7)	137	(17.4)		48	(15.0)	65	(17.4)	
T4	194	(26.3)	233	(29.6)		73	(22.7)	105	(28.1)	
Unknown	18	(2.4)	18	(2.3)		9	(2.8)	9	(2.4)	
N stage										
N0	135	(18.3)	115	(14.6)		63	(19.6)	58	(15.5)	
N1	272	(36.9)	279	(35.4)	0.219	111	(34.6)	140	(37.4)	0.340
N2	146	(19.8)	167	(21.2)		71	(22.1)	73	(19.5)	
N3	171	(23.2)	210	(26.6)		66	(20.6)	94	(25.1)	
Unknown	14	(1.9)	17	(2.2)		10	(3.1)	9	(2.4)	
Histology										
Invasive ductal carcinoma	545	(73.8)	603	(76.5)		234	(72.9)	289	(77.3)	
Invasive lobular carcinoma	81	(11.0)	69	(8.8)	0.506	35	(10.9)	34	(9.1)	0.452
Others	100	(13.6)	104	(13.2)		45	(14.0)	47	(12.6)	
Unknown	12	(1.6)	12	(1.5)		7	(2.2)	4	(1.1)	
Grade										
Well differentiated	48	(6.5)	51	(6.5)		24	(7.5)	32	(8.6)	
Moderately differentiated	251	(34.0)	274	(34.8)	0.935	115	(35.8)	153	(40.9)	0.214
Poorly differentiated	395	(53.5)	419	(53.2)		162	(50.5)	169	(45.2)	
Undifferentiated	7	(0.9)	10	(1.3)		1	(0.3)	5	(1.3)	
Unknown	37	(5.0)	34	(4.3)		19	(5.9)	15	(4.0)	
IHC subtype										
HR+/HER2-	409	(55.4)	446	(56.6)		191	(59.5)	231	(61.8)	
HR+/HER2+	114	(15.4)	147	(18.7)	0.252	55	(17.1)	64	(17.1)	0.941
HR-/HER2+	64	(8.7)	56	(7.1)		28	(8.7)	29	(7.8)	
HR-/HER2-	111	(15.0)	99	(12.6)		24	(7.5)	28	(7.5)	
Unknown	40	(5.4)	40	(5.1)		23	(7.2)	22	(5.9)	
Bone metastasis										
No	263	(35.6)	303	(38.5)		104	(32.4)	144	(38.5)	
Yes	475	(64.4)	485	(61.5)	0.255	217	(67.6)	230	(61.5)	0.094
Lung metastasis										
No	624	(84.6)	654	(83.0)		268	(83.5)	315	(84.2)	
Yes	114	(15.4)	134	(17.0)	0.410	53	(16.5)	59	(15.8)	0.793
Liver metastasis										
No	621	(84.1)	668	(84.8)		282	(87.9)	320	(85.6)	
Yes	117	(15.9)	120	(15.2)	0.736	39	(12.1)	54	(14.4)	0.377
Combination of metastatic sites										
Bone metastasis only	374	(50.7)	380	(48.2)	0.338	173	(53.9)	181	(48.4)	0.148
Lung metastasis only	57	(7.7)	66	(8.4)	0.640	24	(7.5)	28	(7.5)	0.996
Liver metastasis only	54	(7.3)	57	(7.2)	0.950	19	(5.9)	26	(7.0)	0.581
Other metastasis only	147	(19.9)	174	(22.1)	0.300	60	(18.7)	88	(23.5)	0.120
Bone and lung metastases	52	(7.0)	62	(7.9)	0.542	28	(8.7)	29	(7.8)	0.643
Bone and liver metastases	58	(7.9)	57	(7.2)	0.644	19	(5.9)	26	(7.0)	0.581
Lung and liver metastases	14	(1.9)	20	(2.5)	0.397	4	(1.2)	8	(2.1)	0.368
Lung and/or liver metastases	217	(29.4)	234	(29.7)	0.901	88	(27.4)	105	(28.1)	0.846
Multiple sites of metastases										
No	632	(85.6)	677	(85.9)		276	(86.0)	323	(86.4)	
Yes	106	(14.4)	111	(14.1)	0.877	45	(14.0)	51	(13.6)	0.884
Surgery										
Breast conserving surgery	256	(34.7)	290	(36.8)		108	(33.6)	143	(38.2)	
Mastectomy	482	(65.3)	498	(63.2)	0.389	213	(66.4)	231	(61.8)	0.209
Insurance										
Uninsured	14	(1.9)	30	(3.8)		4	(1.2)	6	(1.6)	
Insured	564	(76.4)	599	(76.0)	0.080	250	(77.9)	297	(79.4)	0.558
Medicaid	149	(20.2)	153	(19.4)		62	(19.3)	69	(18.4)	
Unknown	11	(1.5)	6	(0.8)		5	(1.6)	2	(0.5)	
Marital status										
Married	351	(47.6)	396	(50.3)		162	(50.5)	188	(50.3)	
Others	350	(47.4)	358	(45.4)	0.524	139	(43.3)	168	(44.9)	0.691
Unknown	37	(5.0)	34	(4.3)		20	(6.2)	18	(4.8)	

Abbreviations: PSM, propensity score matching; HR, hormone receptor; HER2, human epidermal growth factor receptor 2.
*Pearson's chi-square test.

Figure S1. Comparison of OS between the no-radiotherapy and radiotherapy groups shows significantly favorable CSS and OS in the radiotherapy group. All survival curves were adjusted using the Cox proportional hazard model. (a) OS in patients who underwent breast-conserving surgery, (b) CSS in patients without lymph node involvement (N0), (c) CSS in patients with lymph node involvement (N1–3), (d) OS in patients who underwent mastectomy, (e) OS in patients who survived 6 months or more from the time of diagnosis, and (f) OS in patients who survived 24 months or more from the time of diagnosis. CSS, cancer-specific survival; OS, overall survival; BCS, breast-conserving surgery; MS, mastectomy; RT, radiotherapy; HR, hazard ratio; CI, confidence interval.

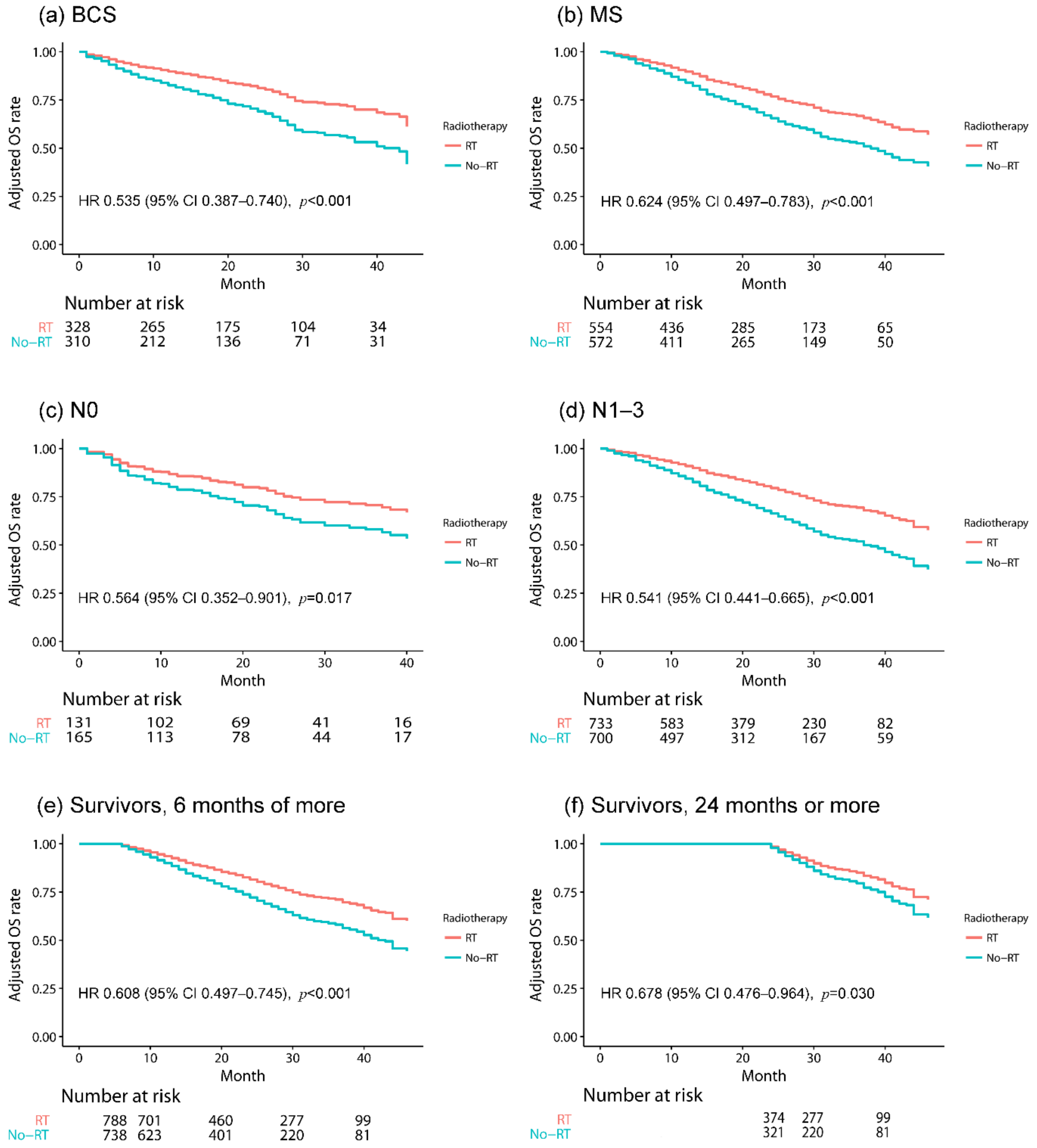

Supplement: Supplementary file 1 — Supplementary tables and figure [file 41598_2019_45016_MOESM1_ESM.pdf]
